# Non-barrier contraceptive use and relation to condom use behaviour by partner type among female sex workers in Andhra Pradesh, India

**DOI:** 10.1136/jfprhc-2014-100918

**Published:** 2015-12-23

**Authors:** Elizabeth Reed, Jennifer Toller Erausquin, Monica Biradavolu, Argentina E Servin, Kim M Blankenship

**Affiliations:** 1 Assistant Professor, Division of Global Public Health, School of Medicine, University of California, San Diego, La Jolla, CA, USA; 2 Assistant Professor, Department of Public Health Education, University of North Carolina at Greensboro, Greensboro, NC, USA; 3 Scholar in Residence, Department of Sociology, American University, Washington, DC, USA; 4 Postdoctoral Fellow, Division of Global Public Health, School of Medicine, University of California, San Diego, La Jolla, CA, USA; 5 Professor and Chair, Department of Sociology, American University, Washington, DC, USA

**Keywords:** condom, family planning service provision, sexually transmitted infections, sex workers, India

## Abstract

**Objective:**

The study assessed non-barrier contraceptive use among female sex workers (FSW) in Andhra Pradesh, India and relation to inconsistent condom use among commercial and non-commercial male sexual partners.

**Methods:**

FSW at least 18 years of age (*n*=2338) were recruited through respondent-driven sampling for an HIV risk survey. Analysis was restricted to women of childbearing age (*n*=2197). Crude and adjusted logistic regression models were used to assess non-barrier contraceptive use and relation to inconsistent condom use with husbands or regular male partners (i.e. non-clients), regular clients and occasional clients.

**Results:**

Non-barrier methods of contraception included contraceptive pills (3.8%) and sterilisation (68.4%). In logistic regression models adjusted for relevant demographics, FSW using contraceptive pills were more likely to report inconsistent condom use with a regular client (past week) [adjusted odds ratio (AOR) 2.2, 95% confidence interval (CI) 1.2–4.0] and with an occasional client (past week) (AOR 2.6, 95% CI 1.6–5.3), as well as accepting more money for sex without a condom (past 30 days) (AOR 2.5, 95% CI 1.5–4.3). No significant associations were found between pill use and inconsistent condom use among women's non-client partners, potentially related to small sample sizes within these subgroups. Reporting sterilisation, which was more common among FSW who were older in age, was not associated with inconsistent condom use with client or non-client sexual partners.

**Conclusions:**

Findings document potential unmet need for modern, spacing contraceptives (i.e. pill, intrauterine device), but also indicate the importance for family planning services, particularly those promoting modern contraceptive methods to be provided alongside HIV prevention among FSW in Andhra Pradesh, India.

**Key message points**

- This study highlights the importance of increasing family planning (FP) service access among female sex workers (FSW) in India.
- FSW who reported contraceptive pill use were more likely to report inconsistent condom use with clients.
- Findings document the importance of integrating FP and HIV prevention services among FSW.

## Introduction

About 2.3 million people are estimated to be living with HIV in India. The southern state of Andhra Pradesh is among the Indian states with the highest rates of infection;^1^ here, the epidemic appears to be largely affecting female sex workers (FSW).^2^ Given the disproportionate burden of HIV among this population, much of the recent public health research and interventions have focused on promoting condom use to prevent HIV.^1–3^

Although the majority of FSW are within reproductive age and also at high risk of unintended pregnancy,^4–9^ family planning (FP) needs of FSW have not been at the forefront of recent research and programming.^10–13^ Previous work among other populations of women has indicated a link between non-barrier contraceptive use and increased inconsistent condom use.^14^ ^15^ Less is known about whether use of non-barrier contraception affects condom use among FSW, particularly in the context of India.^5^ ^10^ Studies have shown that condom use among FSW often varies by partner type; women are less likely to use condoms with relationship partners and regular clients compared to new or occasional clients.^5^ ^16–22^ Various other factors related to women's sex work also contribute to non-condom use among FSW; for example, many FSW make more money for sex trades with no condom used, but also dire economic situations (e.g. to pay debts or difficulties in providing basic necessities for children and household) reported by women increase the urgency of FSW's work and have been associated with reduced condom negotiating power with clients.^8^ ^13^ ^23–26^

Given the high risk for both HIV and unintended pregnancy among FSW,^3–9^ more research is needed on contraceptive use among FSW and whether it is associated with inconsistent condom use. In India, sterilisation is a predominant FP method; in particular, women undergo sterilisation after attaining their desired family size.^27^ However, increasingly, programmes have been developed in India to promote other forms of reversible FP methods in order to improve maternal and child health (e.g. by improving birth spacing and reducing rapid repeat pregnancies).^5^ ^28–30^ Thus, more work is needed to examine modern non-barrier contraceptive use among FSW and potential association with inconsistent condom use with clients and other sex partners. Accordingly, the objective of this study was to assess non-barrier contraceptive use and association with inconsistent condom use among male relationship partners and clients among FSW in Andhra Pradesh, India.

## Methods

### Procedure and sample

Data for this analysis were collected as part of Project Parivartan, which analyses the implementation and impact of a community mobilisation intervention targeted to reduce HIV risk among FSW in Rajahmundry, within the East Godavari District of Andhra Pradesh, India. Quantitative data are from the three rounds of a survey administered among FSW between 2006 and 2010. Participants were at least 18 years of age, female, reported having sex in exchange for money in the year prior to the survey, and were capable of providing informed consent. Participants were recruited via respondent-driven sampling (RDS). Given the high number of waves (i.e. initial participants recruited women who recruited more women and this continued for multiple waves), it is likely that each round represented adequate coverage of the population of FSW in this region.^31^ ^32^ A total of 2338 FSW gave informed consent and completed the survey across all three rounds. For the present analysis, the sample was further restricted to women of childbearing age (18–49 years) (*n*=2197).

Study staff and trained female Telugu-speaking research assistants obtained informed consent and administered the surveys in a Project Parivartan field office. Survey completion time for participants averaged between 90 and 120 minutes. Participants were compensated for their time and costs associated with transportation in accordance with local norms. Survey participants earned an additional incentive payment for each coupon that resulted in the successful recruitment of new study participants. The RDS recruitment process and study protocols have been described elsewhere.^24–26^ ^33^ This research was approved by the Institutional Review Boards at American University and at Duke University, the Human Investigations Committee at Yale University, and the VHS-YRG Care Medical Centre Institutional Review Board in Chennai, India.

### Measures

#### Demographic variables

*Age* was measured continuously and grouped into five categories (18–24, 25–29, 30–34, 35–39, 40–49 years). *Education* was measured according to whether or not women reported having primary education or beyond. *Relationship status* included measures of marital status (currently married, never married, divorced/separated/widowed) as well as whether participants reported currently having a boyfriend or other male sexual relationship partner. *Debt* (whether participants reported current debt/loans) was also assessed. FSW were asked to report the ‘type’ of sex work they practised (*venue*) (brothel, street, lodge or hotel, agricultural fields, home, highway or other). Participants were asked whether they have *children* and the number they have.

#### Non-barrier contraceptive use

One questionnaire item asked women to select which modern contraceptive methods they currently use to protect themselves from pregnancy [condom, pill, sterilisation, injectable contraceptive, intrauterine device (IUD)]. Dichotomous variables were created for each type of non-barrier method reported by women (only pill use and sterilisation were reported).

#### Condom use behaviour

The outcomes for this study were measures of condom behaviour. *Inconsistent condom use* was measured by asking participants how often (five-point Likert scale ranging from ‘never’ to ‘always’) in the past week they have had sex without a condom with a regular client, an occasional client, or a husband or regular partner (e.g. boyfriend); those women who did not report ‘always’ using a condom with these partners in the past week were categorised as having inconsistent condom use. *Accepting more money for unprotected sex* was measured by asking participants if they had accepted more money for sex with no condom in the past 30 days.

### Data analyses

Sample characteristics (e.g. age, debt, education, venue of work, marital and relationship status, and children) were analysed to identify factors that differed between women reporting and not reporting non-barrier contraceptive use. Because women reporting sterilisation specifically were significantly different from other women in several key characteristics, separate regression analyses were conducted for each type of non-barrier contraception reported by women (i.e. pill users and sterilised women were analysed in distinct regression models). Crude and adjusted logistic regression models were used to assess the relation between the use of non-barrier contraceptive methods and (1) women's reports of inconsistent condom use (three separate models for condom use behaviour with regular clients, occasional clients, and husbands or other male relationship partners) and (2) women's reports of accepting more money for unprotected sex. All adjusted models were controlled for the study wave as well as sample characteristics significantly associated (*p*<0.05) with contraceptive use, namely age, marital status, and number of children. For logistic regression findings, odds ratios (ORs) were presented with associated 95% confidence intervals (CIs), and the significance of individual variables was evaluated using Wald Chi-square (χ^2^) tests. All analyses were conducted using SAS V.9.1 (SAS Institute Inc., Cary, NC, USA). RDS weights were not employed, consistent with the authors’ own previously published work using these data as well as published guidelines and methodologies used in other similar studies using RDS.^2^ ^24–26^ ^31–33^ The use of RDS weights makes the greatest difference when responses to questions vary by respondents’ network size. In other analyses from this project, our outcomes of interest (including inconsistent condom use) did not vary significantly based on respondents’ network size.

## Results

### Sample characteristics

Women reported two types of non-barrier contraception methods to prevent pregnancy: contraceptive pills (3.8%) and sterilisation (68.0%). Notably, among those not reporting sterilisation (*n*=694), 12.1% reported contraceptive pill use.

The median age of women in the sample was 30 years (±7.4 standard deviation. Most women (86%) did not report education. About one-quarter (24%) of the sample reported working at home, one-fifth (20%) reported working on the highway, 16% reported working in agricultural fields, 11% reported working on the street, 7% in brothels, 4% in a hotel or lodge, and 19% reported working in other venues. Few (14%) women reported being currently married and 9% said they have another male relationship partner or boyfriend. The majority (90%) of women reported having children; about one-quarter had one child, almost half (47%) had two, and 19% had three or more children (Table 1).

**Table 1:** Sample characteristics (*n*=2197)

Sample characteristics	Total sample (*n*=2197)	Pill use (3.8%, *n*=84)	Sterilisation as contraception (68.4%, *n*=1502)	No contraception (27.8%, *n*=611)
	[% (*n*)]	[% (*n*)]	[% (*n*)]	[% (*n*)]
Age* (years)				
18–24	16.8 (370)	32.1 (27)	10.6 (159)	30.1 (184)
25–29	22.8 (500)	21.4 (18)	23.6 (354)	21.0 (128)
30–34	19.5 (429)	19.1 (16)	21.2 (318)	15.6 (95)
35–39	23.4 (515)	11.9 (10)	26.3 (395)	18.0 (110)
40–49	17.4 (383)	15.5 (13)	18.4 (276)	15.4 (94)
Education				
Less than primary	85.9 (1887)	78.6 (66)	86.2 (1294)	86.3 (527)
Primary	12.9 (284)	20.2 (17)	13.0 (195)	11.8 (72)
College/technical training	1.2 (26)	1.2 (1)	1.0 (13)	2.0 (12)
Debt				
Yes	82.1 (1803)	76.2 (64)	82.7 (1242)	81.3 (497)
No	17.9 (394)	23.8 (20)	17.3 (260)	18.7 (114)
Children (*n*)*				
0	10.0 (220)	14.3 (12)	2.5 (38)	27.8 (170)
1	24.1 (529)	33.3 (28)	18.7 (280)	36.2 (221)
2	46.5 (1021)	34.5 (29)	55.1 (827)	27.0 (165)
3+	19.4 (426)	17.9 (15)	23.7 (356)	9.0 (55)
Relationship status†				
Marital status*				
Married	14.0 (307)	11.9 (10)	15.0 (225)	11.8 (72)
Never married	7.4 (163)	10.7 (9)	5.6 (84)	11.5 (70)
Separated/divorced/widowed	41.8 (919)	50.0 (42)	41.1 (617)	42.6 (260)
Have a boyfriend	9.1 (200)	15.5 (13)	9.2 (138)	8.0 (49)
Venue of work†				
Home	24.0 (525)	27.4 (23)	23.5 (352)	24.7 (150)
Highway	19.5 (426)	16.7 (14)	20.0 (299)	18.6 (113)
Street	11.0 (241)	15.5 (13)	10.8 (162)	10.9 (66)
Brothel	6.9 (151)	7.1 (6)	6.3 (94)	8.4 (51)
Lodge or hotel	4.1 (89)	7.1 (6)	3.9 (59)	4.0 (24)
Agricultural field	15.8 (347)	9.5 (8)	16.8 (252)	14.3 (87)
Other	18.8 (411)	16.7 (14)	18.8 (281)	19.1 (116)

*p<0.05.

†Not mutually exclusive categories.

There were some differences in sample characteristics according to whether women reported pill use, sterilisation or no contraception use. A greater proportion of those women who reported contraceptive pill use or sterilisation reported having children compared to those who did not report contraceptive use (χ^2^=462.2, *p*<0.0001). The type of non-barrier contraception reported also varied by age (χ^2^=139.0, *p*<0.0001) and having ever been married (χ^2^=23.1, *p*<0.0001. Specifically, data suggest that women reporting sterilisation were older and more likely to report having ever been married compared to women reporting pill use or no contraception. Women did not vary significantly based on other sample characteristics.

### Non-barrier contraceptive use and relation to inconsistent condom use

Among FSW who did not report sterilisation, in adjusted logistic regression models (adjusted for age, marital status, and number of children), FSW who reported contraceptive pill use were more likely to report inconsistent condom use with a regular client (past week) (AOR 2.2, 95% CI 1.2–4.0), inconsistent condom use with an occasional client (past week) (AOR 2.6, 95% CI 1.6–5.3) and accepting more money for sex without a condom (past 30 days) (AOR 2.5, 95% CI 1.5–4.3). No statistically significant associations were found between pill use and inconsistent condom use with women's relationship partners (i.e. non-clients) (Table 2). Among women reporting sterilisation, no significant differences were found in inconsistent condom use across partner types compared to women who did not report sterilisation or pill use (the AORs ranged between 1.4 and 1.5; data are not shown in Tables 1 and 2.) However, given that women who reported being sterilised were older than non-sterilised women (which may affect condom use), we conducted exploratory analyses among women in the sample aged ≤30 years. Based on these exploratory analyses, we found that women (aged ≤30 years) who reported sterilisation (compared to those who reported neither sterilisation nor pill use) were 50% more likely to report taking more money for sex with no condom used in the past 30 days (AOR 1.5, 95% CI 1.1–2.2). We did not find any significant associations with other measures of condom use among this age group of women who reported sterilisation.

**Table 2:** The association between women's reports of contraceptive pill use and recent condom behaviour by partner type (*n*=694)

Outcome variable	Total (among those not reporting sterilisation) (*n*=694) [% (*n*)]	Pill use (*n*=84) [% (*n*)]	No pill use (*n*=610) [% (*n*)]	Crude OR (95% CI)*	AOR† (95% CI)
Inconsistent condom use with a regular client (past week) (*n*=553)‡					
Yes	14.8 (82)	25.0 (18)	13.3 (64)	2.2 (1.2–3.9)	2.2 (1.2–4.0)
No	85.2 (471)	75.0 (54)	86.7 (417)	1.0 Ref	1.0 Ref
Inconsistent condom use with an occasional client (past week) (*n*=617)‡					
Yes	11.7 (72)	24.0 (18)	10.0 (54)	2.8 (1.6–5.2)	2.6 (1.6–5.3)
No	88.3 (545)	76.0 (57)	90.0 (488)	1.0 Ref	1.0 Ref
Inconsistent condom use with a husband or relationship partner (past week) (*n*=136)‡					
Yes	54.4 (74)	63.6 (14)	52.6 (60)	1.6 (0.6–4.1)	1.1 (0.5–2.1)
No	45.6 (62)	36.4 (8)	47.4 (54)	1.0 Ref	1.0 Ref
Accepting more money for sex without a condom (past 30 days)					
Yes	15.6 (108)	27.4 (23)	13.9 (85)	2.4 (1.4–4.0)	2.5 (1.5–4.3)
No	84.4 (586)	72.6 (61)	86.1 (525)	1.0 Ref	1.0 Ref

*Author to provide definition for asterisk symbol.

†Adjusted for study wave, age, marital status (married, never married or separated/divorced/widowed) and number of children.

‡These analyses were conducted only among women who reported having these partner types.

AOR, adjusted odds ratio; CI, confidence interval; OR, odds ratio; Ref, referent.

## Discussion

Our findings contribute to the limited body of research that has focused on FP needs among FSW, and the intersection of FP and HIV risk, particularly in India.^5–8^ ^10–13^ Findings highlight low prevalence and low variability of non-barrier contraceptive use (i.e. only contraceptive pill use was reported apart from sterilisation), and the potential unmet need for modern FP methods, particularly those that are female-controlled and reversible (e.g. those that allow for birth spacing and that reduce rapid repeat pregnancies) among this population of FSW in south India. The findings also reveal an association between non-barrier contraceptive use and inconsistent condom use with clients among women who are not sterilised. Most noteworthy, non-barrier contraceptive use appears to be associated with accepting more money for sex trades with no condom used.

The link between contraceptive pill use and condom use behaviour with clients is consistent with the limited research on this topic.^4–8^ While one previous study did not find a significant association between use of non-barrier contraception and condom use with clients among FSW,^17^ it was conducted in Swaziland, where HIV is a more generalised epidemic and thus condom use may be relatively more standard in the context of sex trades compared to India. To our knowledge, the present study is the first to document the link between non-barrier contraceptive use and accepting more money for sex trades with no condom used. Such findings suggest that greater cash returns, together with reduced concerns regarding pregnancy, may be a mechanism that drives the association between non-barrier contraceptive use and inconsistent condom use with clients among FSW. Notably, while household debt (an indicator of economic vulnerability) did not vary significantly by contraceptive use, there was little variability in this sample (81% of respondents reported debt); this may have masked any mediational effects.

We did not find any significant associations between sterilisation and inconsistent condom use across partner types. Notably, women who are older are also more likely to report accepting more money for sex without a condom and to report inconsistent condom use generally; and thus when we restricted our analyses to women reporting sterilisation, we were also restricting the analyses to an older age group of women who were more likely to report inconsistent condom use. However, we found in exploratory analyses that compared to women who reported no sterilisation, women aged ≤30 years who reported sterilisation were 50% more likely to report taking more money for sex with no condom used in the past 30 days. While sterilisation was not associated with other inconsistent condom use-related outcomes across partner types among this younger age group, these exploratory findings also support the hypothesis that greater cash returns may be a primary mechanism that explains the associations between non-barrier contraceptive use and inconsistent condom use with clients.

Most previous studies investigating the effect of contraceptive use on condom use among FSW have focused only on commercial partners. To our knowledge, only one study among FSW in Bolivia found non-barrier contraceptive use to be significantly related to decreased condom use with non-client sex partners.^16^ In our sample, there were already high rates of unprotected sex with non-commercial partners, as is consistent with previous studies.^34^ ^35^ Previous work has suggested that among FSW, non-condom use with non-commercial partners is primarily a means of establishing trust and intimacy.^21^ ^36^ ^37^ Additionally, small sample numbers may have resulted in limited statistical power to detect a significant association.

Given the low rates of pill use and lack of use of other forms of reversible contraception (e.g. IUDs, injectable contraception, etc.), our findings also suggest that FSW may face unmet need for non-barrier contraceptive methods apart from sterilisation. Non-barrier contraceptive methods are reported to have a prevalence of between 6% and 9% among other populations of women from this region^38^ (compared to 3% in our sample). Furthermore, national data from India indicate that 37% of women of childbearing age report using sterilisation as the main form of contraception, compared to 71% of our FSW sample.^5^ ^28–30^ FSW face high rates of stigma and discrimination and thus may have greater barriers to accessing these other contraceptive methods.^24^ ^39^ ^40^ Our previous work documented that high demands related to caretaking of children places FSW at increased vulnerability to HIV/STI.^26^ Many FSW reported that sex work is a means to financially provide for their children, with the majority also reporting no other financial support from male partners or fathers of their children.^26^ Thus women's control over, and choice regarding FP is an especially important consideration; and in this population of FSW, unmet need for FP is likely to be one factor underlying women's involvement in sex work. Taken together, these findings indicate the critical need to ensure access to FP services as well as HIV prevention programmes among FSW, and that these services need to be well-integrated to promote ‘dual protection’ (i.e. the use of both condoms and non-barrier contraceptive methods).

The present study has several limitations. The cross-sectional design does not establish the temporality of these associations. Additionally, the items used for analyses rely on self-reported responses. Stigma can often result in under-reporting of sensitive issues or socially undesirable behaviours, such as inconsistent condom use.^41–43^ However, such under-reporting would decrease the power to detect an association between contraceptive pill use and inconsistent condom use. The present study found multiple strong links among these factors. Also in terms of variables measured, there may have been confounders in the association between contraceptive pill use and condom use that this study did not measure. We did not measure or test for HIV, which may be associated with condom use as well as contraception. Regarding study recruitment, while we cannot guarantee that we reached all groups or acquired a representative sample of the underlying population, previous studies have found RDS to be the most effective method for sampling ‘hard to reach’ populations, including FSW.^44–47^ Based on the high number of recruitment waves following the initial seed for each survey round, we are confident that we achieved a diverse sample of FSW in the study areas. As we included data from three rounds of data collection in the current analyses, there is a possibility that some women may have participated more than once. To address this possibility, we conducted a separate exploratory analysis that deleted all women who reported prior participation in the study from subsequent study waves, and we did not find any noteworthy differences in the findings. Finally, the present study findings are most applicable to FSW populations working in Rajahmundry, Andhra Pradesh and may not be generalisable to other FSW populations from this state or other Indian states (or indeed elsewhere).

These limitations notwithstanding, our findings highlight the low prevalence of non-barrier contraceptive use (aside from sterilisation) and likely unmet need for modern spacing methods of FP methods among FSW. Given the link between contraceptive pill use and reduced condom use with clients among FSW in this sample, the findings support a need for integrated HIV prevention and FP programmes among FSW. These findings are especially critical given increasing efforts to promote contraceptive methods other than sterilisation, such as the pill, to promote birth spacing in this setting. To the extent that the association between contraceptive pill use and non-condom use can be explained by the greater cash returns of non-condom use, as well as reduced pregnancy concerns, such integrated programmes may also need to address women's economic vulnerability.
